# Morbidity and Mortality According to Latest CD4+ Cell Count among HIV Positive Individuals in South Africa Who Enrolled in Project Phidisa

**DOI:** 10.1371/journal.pone.0121843

**Published:** 2015-04-09

**Authors:** Patrick H. Maduna, Matt Dolan, Lwando Kondlo, Honey Mabuza, Judith N. Dlamini, Mike Polis, Thabo Mnisi, Susan Orsega, Patrick Maja, Lotty Ledwaba, Thuthukile Molefe, Phumelele Sangweni, Lisette Malan, Gugu Matchaba, Paul Khabo, Greg Grandits, James D. Neaton

**Affiliations:** 1 South Africa Military Health Services, South African National Defence Forces, Pretoria, South Africa; 2 The Henry M. Jackson Foundation for the Advancement of Military Medicine, Inc., Bethesda, Maryland, United States of America; 3 Charisma Healthcare Solutions, Pretoria, South Africa; 4 National Institute of Allergy and Infectious Diseases, National Institutes of Health, Bethesda, Maryland, United States of America; 5 Division of Biostatistics, University of Minnesota, Minneapolis, Minnesota, United States of America; Rega Institute for Medical Research, BELGIUM

## Abstract

**Background:**

Short-term morbidity and mortality rates for HIV positive soldiers in the South African National Defence Force (SANDF) would inform decisions about deployment and HIV disease management. Risks were determined according to the latest CD4+ cell count and use of antiretroviral therapy (ART) for HIV positive individuals in the SANDF and their dependents.

**Methods and Findings:**

A total of 7,114 participants were enrolled and followed for mortality over a median of 4.7 years (IQR: 1.9, 7.1 years). For a planned subset (5,976), progression of disease (POD) and grade 4, potentially life-threatening events were also ascertained. CD4+ count and viral load were measured every 3 to 6 months. Poisson regression was used to compare event rates by latest CD4+ count (<50, 50–99, 100–199, 200–349, 350–499, 500+) with a focus on upper three strata, and to estimate relative risks (RRs) (ART/no ART). Median entry CD4+ was 207 cells/mm^3^. During follow-up over 70% were prescribed ART. Over follow-up 1,226 participants died; rates ranged from 57.6 (< 50 cells) to 0.8 (500+ cells) per 100 person years (py). Compared to those with latest CD4+ 200–349 (2.2/100py), death rates were significantly lower (p<0.001), as expected, for those with 350–499 (0.9/100py) and with 500+ cells (0.8/100py). The composite outcome of death, POD or grade 4 events occurred in 2,302 participants (4,045 events); rates were similar in higher CD4+ count strata (9.4 for 350–499 and 7.9 for 500+ cells) and lower than those with counts 200–349 cells (13.5) (p<0.001). For those with latest CD4+ 350+ cells, 63% of the composite outcomes (680 of 1,074) were grade 4 events.

**Conclusion:**

Rates of morbidity and mortality are lowest among those with CD4+ count of 350 or higher and rates do not differ for those with counts of 350–499 versus 500+ cells. Grade 4 events are the predominant morbidity for participants with CD4+ counts of 350+ cells.

## Introduction

South Africa is one of the first nations to integrate persons with HIV into military deployments and peacekeeping operations. When deciding whether an HIV positive participant should be included as part of internal or external military deployments, reliable quantification of the risk of serious illness over the next 6–12 months is necessary [1].

CD4+ cell count is an established prognostic marker for AIDS and death [2], and current/most recent CD4+ cell count has been shown to be a good predictor of short-term risk of AIDS and death in cohorts comprised of participants in both resource-rich and resource-poor settings [3–12]. Such data for ART-naïve patients have been used to make decisions about when to initiate antiretroviral treatment [3]. Until recently, there were less data on risks of non-AIDS events such as major cardiovascular, renal and liver disease events and non-AIDS cancer [13–17]. These events have been associated with HIV disease and ART [18] and may be associated with a greater risk of death than AIDS events [19], therefore, an overall assessment of the short-term risk of serious illness needs to also consider non-AIDS conditions. With few exceptions [7, 20], most data on serious non-AIDS conditions have been generated from cohorts in high-income countries and, as noted in a recent editorial, the spectrum of illness among individuals with HIV in Africa is likely different [21].

Many studies involving African cohorts have also had high lost to follow-up rates [12, 22–24]. Larger cohorts with follow-up for mortality and different types of morbidity are needed to inform policy on military deployments in South Africa. Such data will also inform the management of HIV patients more generally with high CD4+ cell counts and help establish future research directions.

In this paper data from a large cohort of over 7,000 HIV positive South African National Defence Force (SANDF) members and their dependents who were followed for several years are used to estimate the risk of death and serious illness according to latest CD4+ cell count and to assess whether those risks vary according to use of ART.

## Methods

### Study population

Project Phidisa is an HIV/AIDS research collaboration between the South African Department of Defence (SA DoD) through the South African Military Health Service (SAMHS), the US Department of Health and Human Services through the National Institutes of Health (NIH), and the US Department of Defense (US DoD). It was established in 2003 with the aim of studying HIV in a sample of SANDF members and their dependents.

Project Phidisa includes participants enrolled in 3 protocols at 6 SAMHS sites: hospitals in Pretoria, Cape Town and Bloemfontein, and sickbays in Phalaborwa, Mtubatuba, and Mthatha. Beginning in January 2004, HIV-infected SANDF members and their dependents could be enrolled in a cohort study (Phidisa 1) or in a randomized trial (Phidisa 2) (ClinicalTrials.gov identifier: NCT00342355) [25]. For Phidisa 1, the inclusion criteria were very broad; any HIV-positive individual in the SANDF or their family members could be enrolled. For Phidisa 2, HIV-positive individuals could be randomized to one of four ART regimens if their CD4+ count was < 200 cells or they had a prior AIDS diagnosis and they met the following other eligibility criteria: hemoglobin level of 9 g/dL or higher (8 g/dL or higher for women), a neutrophil count > 500 cells/μL, a platelet count > 25,000 platelets/μL, and a serum liver transaminase level < 5 times the upper limit of the normal range [25]. Participants who were not enrolled in the trial received ART according to South African national guidelines [26].

In March 2008, after the clinical trial was completed, all participants in Phidisa 1 and Phidisa 2 were invited to participate in an amended observational study protocol (Phidisa 1a). Phidisa 1a included participants in Phidisa 1 and Phidisa 2 who reconsented and newly enrolled HIV positive participants. Like Phidisa 1, the inclusion criteria were very broad, any HIV-positive SANDF member or their family members could be enrolled, and for ART-naïve participants, ART was initiated according to South African national guidelines [26]. Phidisa 1 and 1a also included participants who were not HIV positive. These participants are not included in this report.

### Ethics statement

The study was approved by the Phidisa/SANDF Institutional Review Board and National Institute of Allergy and Infectious Diseases (NIAID) Institutional Review Board. Written informed consent was obtained from all participants.

### Data collection and follow-up of participants

At the time of enrollment (baseline), a medical history was obtained, a physical examination was performed, and plasma HIV RNA level, CD4+ cell count, hematology and clinical chemistries were determined. The database does not include information to identify a SANDF member from a dependent.

Prior to April 2008, follow-up visits occurred at least every 3 months for trial participants (Phidisa 2) and at least every 6 months for participants who were not in the trial (Phidisa 1). After April 2008 and through February 2012, participants in Phidisa 1a were seen every 3 months if on ART and every 6 months if not on ART. In 2012, the follow-up visit schedule for all participants was changed to every 6 months.

At each follow-up visit, interim medical and treatment histories were obtained and CD4+ cell counts and HIV RNA levels were measured. Deaths were reported throughout foIlow-up for participants in Phidisa 1, Phidisa 2, and Phidisa 1a. Henceforth, this is referred to as the mortality cohort. Follow-up time for this cohort began at enrollment date into Phidisa 1, Phidisa 2, or Phidisa 1A. POD events, which include AIDS-defining events and pulmonary tuberculosis (TB), and events considered potentially life-threatening (grade 4 events), which were not limited to laboratory abnormalities, were collected for participants in Phidisa 2 and for all participants enrolled in Phidisa 1a. Henceforth, this subset of participants is referred to as the morbidity and mortality cohort. Follow-up time for this cohort began at enrollment date into Phidisa 2 or Phidisa 1A.

Grade 4 events were coded according to the *Medical Dictionary for Regulatory Activities* (MedDRA) (version 12.0). MedDRA codes corresponding to myocardial infarction, stroke, end-stage renal disease, cirrhosis, and non-AIDS cancer, excluding skin neoplasms (serious non-AIDS conditions) are summarized as well as grade 4 events classified according to 26 different system organ classes (SOCs).

For participants who enrolled in Phidisa 1a follow-up continued through February 28, 2013 except those enrolled in satellite sites of Mtubatuba and Mthatha, and for those enrolled at Phalaborwa. Follow-up ended for participants at the satellite sites on June 1, 2012 and for the participants at Phalaborwa on January 31, 2013. After the end of follow-up sites were asked to verify vital status, POD and grade 4 event status as of their site closing date for all participants who had not been seen for 12 months. For many participants who had been seen in the last 12 months, vital status and POD and grade 4 event status was also verified as of the closing date for the site. For each participant the date last known to be alive and date last known to be free of POD or grade 4 events were recorded. Participants who were not seen in the 12 months prior to the closing date for the site were considered lost to follow-up for vital status (or POD or grade 4 events).

### Statistical analysis

For the mortality cohort, death rates were calculated by accumulating person time according to latest CD4+ cell count (<50, 50–99, 100–199, 200–349, 350–499 and 500+ cells). Person time was counted from the time of each CD4+ measurement until the first of the following: next CD4+ cell count, death, date last known alive, or date of closeout. CD4+ cell counts were updated every 3 or 6 months; thus, the rates can be interpreted as the short-term risk of death associated with a given CD4+ cell count, a measure that was considered to be relevant for making decisions about deployment and clinical management. For analyses according to use of ART, follow-up was censored for the cohort not on ART when ART was initiated. For these analyses, once a person started ART they were considered always on ART. The number of events and rates per 100 person years (PY) are cited. Standard errors of the rates can be obtained by dividing the rate by the square root of the number of events. Poisson regression (using the GENMOD procedure in SAS) with log person years as an offset and a compound symmetry covariance structure to account for the repeated CD4+ periods within a person was used to determine unadjusted and adjusted relative rates (RR) and 95% confidence intervals (CIs). CD4+ was modeled as both a continuous variable (square root transformation) to assess trends and as a model with 5 indicators variables to represent the six CD4+ strata. For the latter, those with latest levels of CD4+ 200–349 cells/mm^3^ were used as the reference group. In addition comparisons between the upper two CD4+ count strata are made (350–499 versus 500+ cells). Adjustment considers age, gender, and history of POD at the time of enrolment, and time-updated HIV viral load categorized as < 400, 400–9999, and 10,000+ copies/mL. Deviance statistics and deletion diagnostics indicated a good fit of the Poisson model [27].

For the morbidity and mortality cohort rates of death, POD and grade 4 events according to the latest CD4+ cell count were computed using the same censoring rules as the mortality cohort. For POD and grade 4 events and for the composite of death, POD and grade 4 events, multiple events were counted. Poisson regression models with continuous CD4+ count after square root transformation and with categorical CD4+ counts were considered. For the latter, the 200–349 CD4+ cell group was used as reference; as for the mortality cohort, comparisons are also made between those with 350–499 and 500+ latest CD4+ cell counts. In addition, within each CD4+ stratum, RRs (ART versus no ART) were estimated using Poisson regression using an indicator variable for ART status. Covariate adjustment was similar to the mortality cohort except that in analyses for those not on ART adjustment was for log_10_ viral load instead of the 3 viral load categories.

For analyses of associations of latest CD4+ cell count with mortality for those on and not on ART, instead of censoring follow-up for those not on ART when ART was initiated, a second analysis was also performed which considered the initiation of ART as a competing risk. For this analysis a competing risk proportional hazards regression model was used [28]. A referee noted that this is a more appropriate estimate than hazards determined from a regression model that considers the cause-specific hazards (e.g., mortality and initiation of ART) because of their dependence (i.e., participants with lower CD4+ counts are more likely to initiate ART and someone who has initiated ART has zero risk of pre-ART death). This dependence would lead to bias in comparing associations between latest CD4+ cell count and mortality for those not on ART and those on ART. For those not on ART, a supplemental table (S3 Table) is included which compares estimates of CD4+ modeled as a continuous variable and with 5 indicators variables as mentioned in the preceding paragraph when a cause-specific hazard proportional hazards regression model is used and when a competing risk proportional hazards regression model. The former is similar to the Poisson regression model described above.

All analyses ignored the treatment assignment in Phidisa 2 since rates of mortality and morbidity did not differ among the four randomized groups [25]. For selected comparisons, analyses are cited for those in the trial separately from those who did not participate in the trial.

All reported P values are 2 sided; difference with P <. 05 were considered significant. SAS (version 9.4, SAS institute) was used.

## Results

**Fig 1:**
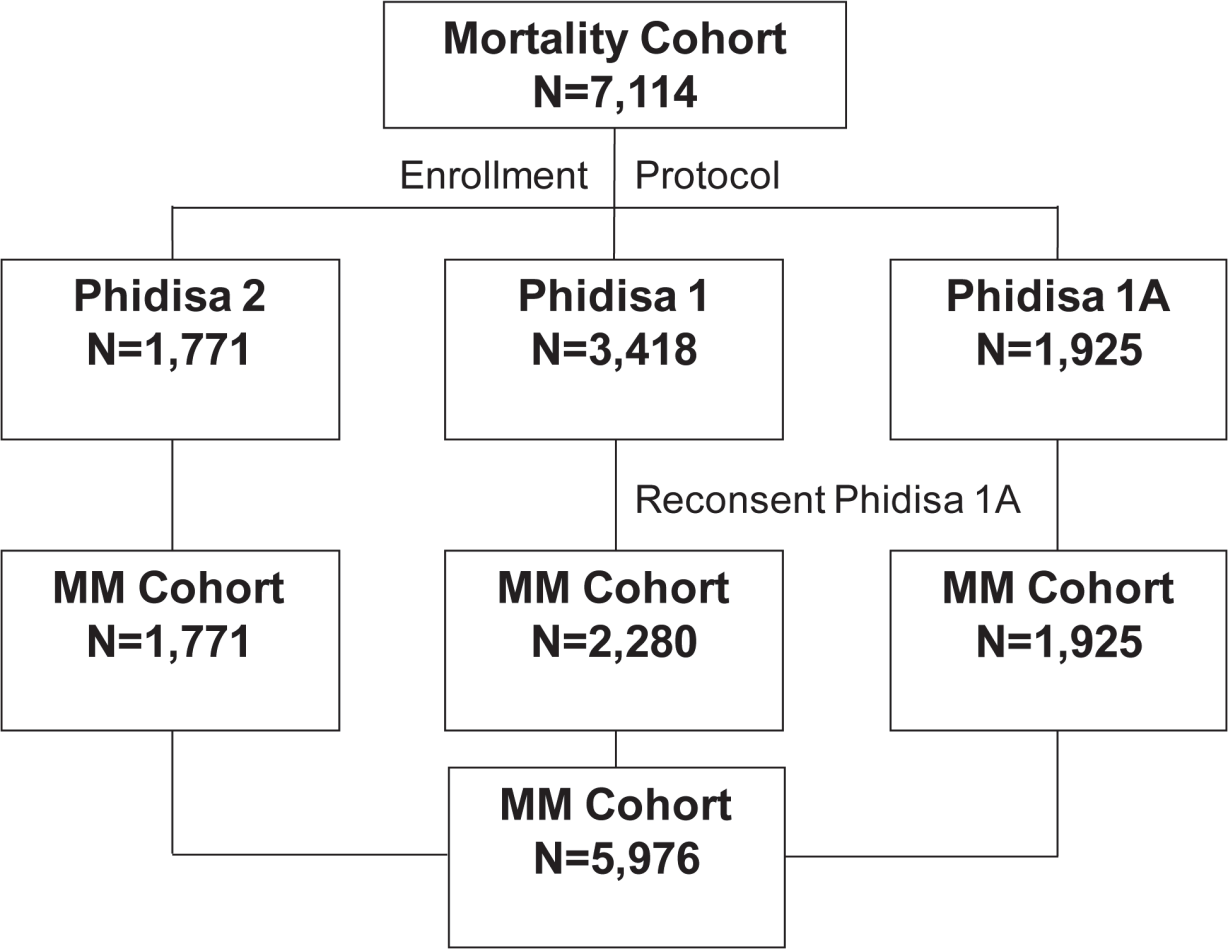
Phidisa Mortality and Morbidity and Mortality (MM) Cohorts. The mortality cohort is made up of 7,114 HIV+ participants: 1,771 enrolled into Phidisa 2 (the clinical trial), 3,418 into Phidisa 1 (cohort study) and 1,925 into Phidisa 1A (cohort study). Of these 7,114 participants, 5,976 participants were in the morbidity and mortality cohort. This includes the 1,771 participants from Phidisa 2, the 1,925 participants from Phidisa 1A and 2,280 participant form Phidisa 1 who reconsented into Phidisa 1A. The participants excluded from the MM cohort are the 1,138 participants enrolled in Phidisa 1 only in which morbidity data was not collected.

**I**n Project Phidisa, 7,114 HIV positive participants were enrolled in the mortality cohort; 3,418 were enrolled in Phidisa 1, 1,771 in Phidisa 2 and 1,925 in Phidisa 1a (Fig 1). At enrolment, median age was 35 years; 37.5% were female; median CD4+ count was 207 cells/mm^3^; 48.4% had CD4+ counts < 200, 25.2% had counts 200–349, and 26.3% had counts of 350 cells or higher; 282 participants (4.4%) were prescribed ART at enrolment (Table 1).

**Table 1:** Characteristics at Enrolment of Phidisa HIV Positive Participants by Mortality Status: Mortality Cohort.

*Demographics*	Known Deceased	Known Alive	Unknown	Total
Age (median years)	35.0 [32.0, 39.0]	35.0 [32.0, 39.0]	33.0 [30.0, 37.0]	35.0 [32.0, 39.0]
Female (%)	279 (22.8%)	2049 (39.7%)	338 (46.7%)	2666 (37.5%)
Location of home (% rural)	501 (40.9%)	2247 (43.5%)	231 (32.0%)	2979 (41.9%)
Marital status (% married)	697 (56.9%)	3474 (67.3%)	456 (63.2%)	4627 (65.1%)
Education (% HS or tertiary)	957 (78.3%)	4183 (81.2%)	623 (86.5%)	5763 (81.2%)
Body Mass Index (median kg/m^2^)	21.2 [18.9, 24.3]	24.3 [21.5, 28.1]	24.3 [21.4, 28.2]	23.8 [20.9, 27.6]
On ART at Baseline (%)	22 (2.3%)	233 (4.8%)	27 (4.5%)	282 (4.4%)
***HIV characteristics***				
CD4 count (median cells/mm^3^)	94.5 [27.0, 231.5]	221.0 [105.0, 372.0]	277.0 [147.0, 444.0]	207.0 [87.0, 360.0]
< 50	432 (35.5%)	663 (12.9%)	59 (8.2%)	1154 (16.3%)
50–99	198 (16.3%)	537 (10.4%)	53 (7.4%)	788 (11.1%)
100–199	216 (17.8%)	1131 (22.0%)	142 (19.7%)	1489 (21.0%)
200–349	222 (18.3%)	1372 (26.7%)	187 (26.0%)	1781 (25.2%)
350–499	83 (6.8%)	726 (14.1%)	148 (20.6%)	957 (13.5%)
500 +	65 (5.3%)	712 (13.8%)	130 (18.1%)	907 (12.8%)
HIV viral load (median log_10_ copies/mL)	5.2 [4.7, 5.6]	4.7 [4.0, 5.2]	4.4 [3.7, 5.1]	4.8 [4.1, 5.3]
Hb (median g/dl)	11.6 [9.8, 13.4]	13.1 [11.6, 14.4]	13.1 [11.5, 14.4]	12.9 [11.3, 14.3]
***Co-morbidities***				
Hepatitis B—SAG positive (%)	66 (5.5%)	179 (3.5%)	12 (1.7%)	257 (3.6%)
Hepatitis C (%)	7 (0.6%)	27 (0.5%)	3 (0.4%)	37 (0.5%)
History of AIDS or Pulmonary/Extrapulmonary TB (%)	415 (43.7%)	1089 (22.4%)	105 (17.7%)	1609 (25.2%)
***Number of patients***	**1226**	**5164**	**724**	**7114**

**Fig 2:**
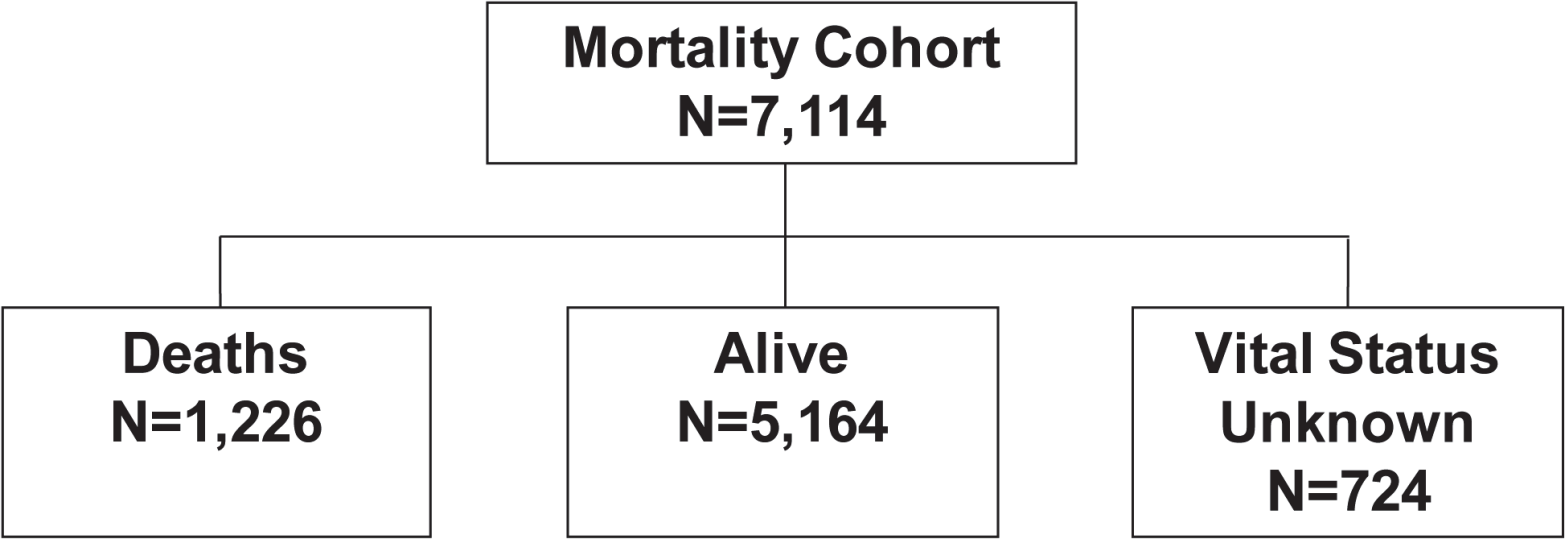
Mortality Status at the End of Follow-up for the Mortality Cohort.

**Fig 3:**
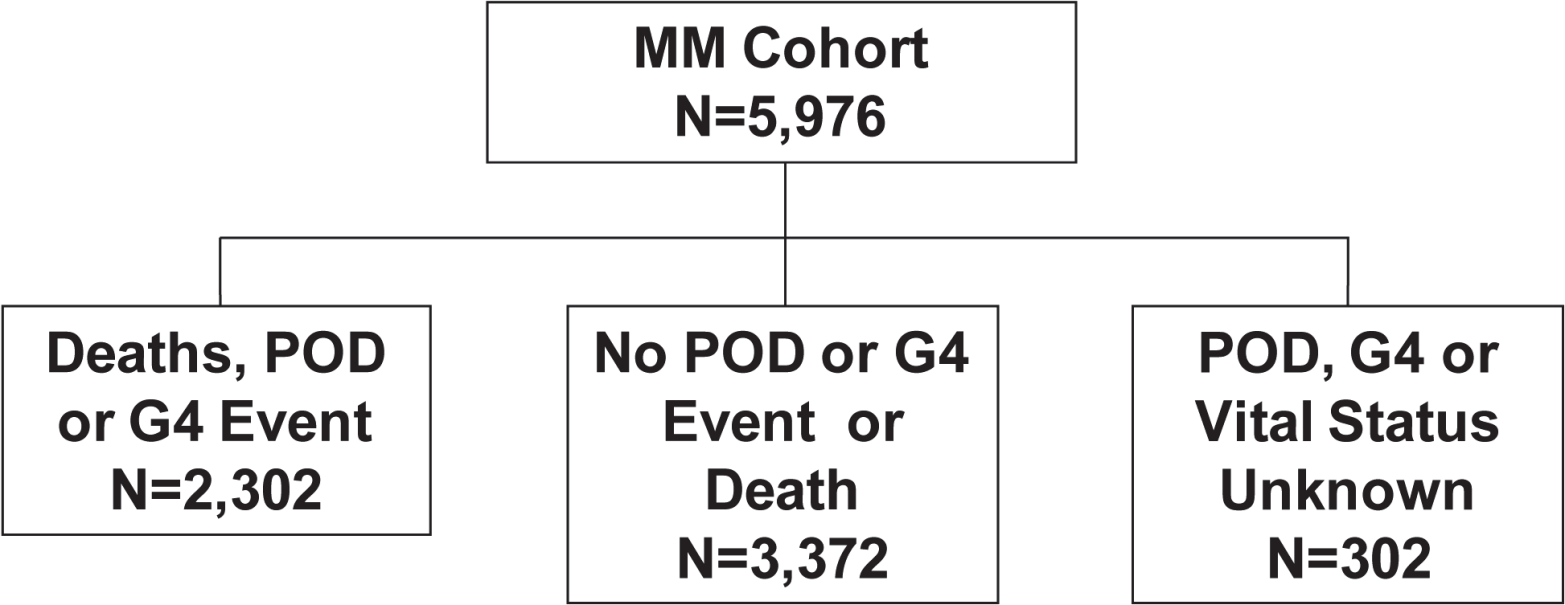
Event Status at the End of Follow-up for the Morbidity and Mortality Cohort.

Enrolment characteristics for the three Phidisa groups which comprise the mortality cohort are given in S1 Table. Of these 7,114 participants, 5,976 participants were in the morbidity and mortality cohort (referred to as MM cohort in Fig 1). Enrolment characteristics for the morbidity and mortality cohort were similar to those for the mortality cohort (see S2 Table). Flow diagrams giving the number of participants who developed an event and the number with unknown event status are shown in Figs 2 and 3.

Median follow-up for participants in the mortality cohort was 4.7 years (IQR: 1.9,7.1 years); 1,226 participants died over a median follow-up of 0.8 years (IQR: 0.2, 2.4 years). A closeout visit form that verified vital status and POD and grade 4 event status as of the site closing date was completed for 93.8% of participants who had not attended a study visit in the 12 months before closure; a closeout form was completed for 80.2% of participants who had been seen within 12 months of site closure. Based on this information, vital status was unknown (i.e., no contact for at least 12 months) for 724 participants (10.2%); using a definition of no contact for 9 months, the percent with unknown vital status is 12.0%. Expressed as a rate, lost to follow-up was 2.2 per 100 person years. Compared to deaths and known survivors, those with unknown vital status were more likely to be female and have higher CD4+ cell counts at entry (Table 1). The 724 participants with no contact for at least 12 months were followed for 1,527 person years before becoming lost-to-follow-up. The median CD4+ cell count at the last follow-up visit they attended was 303 cells (IQR: 117 to 452).

Compared to participants alive at the end of follow-up, participants who died were more likely to be male (77.2% versus 60.3%), and had lower BMI (21.2 versus 24.3 kg/m^2^) and CD4+ cell counts (94.5 versus 221 cells/mm^3^; 35.5% of patients who died had a CD4+ count at enrolment < 50 cells as compared to 12.9% of survivors). Those who died had higher HIV RNA levels at enrolment (5.2 versus 4.7 log_10_ copies/mL), were less likely to be on ART (2.3% versus 4.8%), and were more likely to have a history of POD (43.7% versus 22.4%).

### Use of ART and CD4+ cell count and HIV RNA measurements during follow-up

Of the 7,114 participants in the mortality cohort 2,003 (28.2%) were prescribed ART by completion of the enrolment visit; 1,771 of these participants were prescribed ART as part of the randomized trial (Phidisa 2). During follow-up another 3,035 of the 7,114 participants (42.7%) initiated ART. Thus, for 70.9% of participants in the mortality cohort ART was prescribed at some point during the follow-up period. In total, for the mortality cohort, 20,734 person years accrued on ART and 11,282 person years accrued not on ART. The median (IQR) CD4+ at the time ART was first prescribed during follow-up was 143 (67, 210). Most initiated ART when their CD4+ count was < 200 cells/mm^3^ (71.5%); 1,042 (21.6%) initiated cART at counts between 200 and 349 cells; 331 (6.9%) initiated at a count > 350 cells/uL. There were 66,007 follow-up visits after initiating ART. At 75.2% of these visits, the HIV RNA level was < 400 copies/mL. The median CD4+ count at study entry for participants who did not initiate ART during follow-up was 335 cells (IQR: 153 to 508). During follow-up, the number of CD4+ counts for each participant ranged from 1 to 36 with a median number of 11 (IQR: 4–19).

These follow-up statistics were similar for the 5,976 participants in the morbidity and mortality cohort. ART was prescribed for 4,999 (83.7%) of these participants at entry or during follow-up; 20,672 person years accrued on ART and 10,236 person years accrued not on ART; and the median CD4+ cell count at the time ART was prescribed during follow-up was 143 cells (IQR: 67, 210). These participants had CD4+ cell counts measured at a median of 14 visits (IQR: 7, 21).

### Death rates by latest CD4+ count in the mortality cohort

The overall death rate was 3.8 per 100 person years (for 6 participants who died a prior CD4+ count were not available and they are excluded). Latest CD4+ cell count specific death rates declined from 57.6 per 100 person years for those in the < 50 cell stratum to 0.8 per 100 person years in the 500 cell and higher CD4+ cell count stratum (Table 2).

**Table 2:** Mortality Rates by Latest CD4 Count (cells/mm^3^) for Phidisa Participants: Mortality Cohort.

	Mortality					
			Unadjusted		Adjusted	
Latest CD4 Count	No. Events	Event Rate Per 100 PY	Relative Rate	P-Value	Relative Rate	P-Value
< 50	423	57.6	26.13 (22.17–30.80)	<.001	12.95 (10.49–15.98)	<.001
50–99	211	20.0	9.10 (7.52–11.00)	<.001	5.84 (4.69–7.28)	<.001
100–199	218	5.4	2.46 (2.05–2.96)	<.001	1.96 (1.60–2.40)	<.001
200–349	230	2.2	1.00 (Reference)		1.00 (Reference)	
350–499	75	0.9	0.43 (0.33–0.56)	<.001	0.44 (0.33–0.58)	<.001
≥ 500	63	0.8	0.37 (0.28–0.49)	<.001	0.44 (0.32–0.60)	<.001
Total (Estimate per sqrt cell)	1220	3.8	-0.248 ± 0.006		-0.197 ± 0.008	

**Notes:** Relative rate estimates obtained using Poisson Regression

Adjusted for age, gender, history of AIDS or TB, and time updated viral load (<400, 400–9999, 10000+)

Eight hundred and fifty-two of the 1,220 deaths (69.8%) occurred when the latest CD4+ cell count was < 200 cells. Compared to those with latest CD4+ counts 200–349 cells, adjusted RRs of death were significantly higher (p<0.001) in each of the three lowest CD4+ strata, and significantly lower (p<0.001) for those with CD4+ counts in two higher strata. Death rates in the upper two CD4+ strata were similar (0.9 and 0.8 per 100 person years). The adjusted RR and 95% CI for mortality while CD4+ count was 500+ versus 350–499 was 1.01 (95% CI: 0.70 to 1.46).

**Fig 4:**
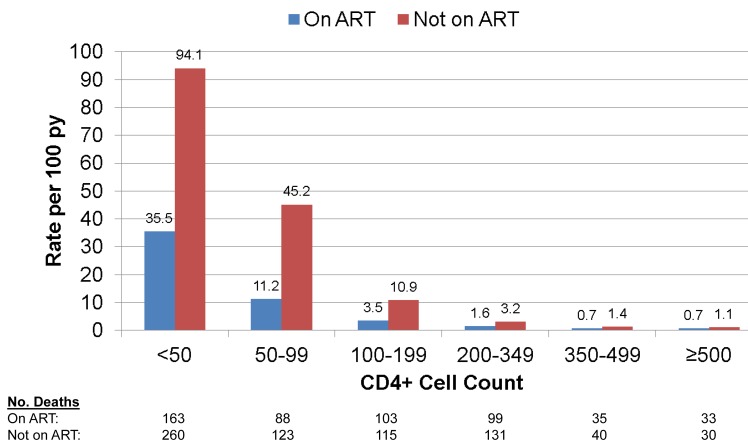
Mortality by Latest CD4+ Cell Count and ART status for Phidisa Participants.

Fig 4 gives death rates by latest CD4+ cell count according to use of ART. In each CD4+ count group, rates of death for those not on ART were greater than for those on ART.

For those on ART, adjusted RRs defined like those in the last column of Table 2 were 16.5 (95% CI: 11.9 to 22.9), 5.7 (95% CI: 4.2 to 7.9), 1.9 (95% CI: 1.4 to 2.5), 0.41 (95% CI: 0.27 to 0.62) and 0.47 (95% CI: 0.31 to 0.70) for those with latest CD4+ cell counts < 50, 50–99, 100–199, 350–499, and 500+, respectively, relative to the group with a latest count of 200–349 cells. We carried out an analysis separately for participants in the Phidisa 2 clinical trial and for those who were on ART but not in the trial. The slope of mortality on latest CD4+ cell count after square root transformation was greater for those in Phidisa 2 (-0.249) compared to those who were not (-0.148). This was due to the much higher death rate in the lower latest CD4+ count group (<50 cells) for trial participants (48.8 per 100 person years) as compared to those who were not in the trial (21.8 per 100 person years). For the other latest CD4+ count groups (50–99, 100–199, 200–349, 350–499 and 500+ cells) death rates were similar for the two cohorts (12.7, 3.8, 1.3, 0.7 and 0.6 per 100 person years for Phidisa 2 participants and 9.8, 3.1, 1.8, 0.7 and 0.8 per 100 person years for those on ART but not in Phidisa 2).

For participants not on ART, the adjusted RRs were 11.0 (95% CI: 8.0 to 15.2), 7.2 (95% CI: 5.2 to 10.0), 2.4 (95% CI: 1.8 to 3.2), 0.48 (95% CI: 0.32 to 0.73) and 0.44 (95% CI: 0.28 to 0.71) for those with latest CD4+ cell counts < 50, 50–99, 100–199, 350–499, and 500+, respectively, relative to the group with a latest count of 200–349 cells.

The CD4+ slopes for those on ART and not on ART were -0.209 (SE = 0.013) and -0.187 (SE = 0.011), respectively (p = 0.20 for difference in slopes). Of the 624 deaths that occurred with latest CD4+ cell counts < 100 cells, 61.4% were patients who died before starting ART.

### Mortality, POD and grade 4 event rates by latest CD4+ count and use of ART in the morbidity and mortality cohort

The morbidity and mortality cohort is comprised of 5,976 participants from 3 groups; 1,771 were enrolled in Phidisa 2 (93% of participants who were alive at the time of enrolment in Phidisa 1a reconsented for further follow-up), 2,280 were initially enrolled in Phidisa 1 and reconsented to participate in Phidisa 1a (for these participants, only follow-up during Phidisa 1a is used), and 1,925 were newly enrolled in Phidisa 1a (Fig 1). Event (death, POD or grade 4) status was unknown (no contact for 12 months) for 302 (5.1%) of the participants in this cohort (Fig 3); this percent was 6.0% if no contact within 9 months is considered. Expressed as a rate, the lost to follow-up was 1.0 per 100 person years.

Table 3 summarizes findings by latest CD4+ cell count for death, POD, grade 4 events and the composite outcome that includes these events. For the composite outcome and each component, rates declined with increasing CD4+ cell count. Adjusted slopes were significantly less than zero for the composite outcome and each component. The most negative slope was found for all-cause mortality (slope = -0.213, SE = 0.011); the least negative was observed for grade 4 events (slope = -0.086, SE = 0.007).

**Table 3:** Mortality and Morbidity Rates by Latest CD4 Count (cells/mm^3^) for Phidisa Participants: Morbidity and Mortality Cohort.

			Unadjusted		Adjusted	
Mortality	No. Events	Event Rate Per 100 PY	Relative Rate	P-Value	Relative Rate	P-Value
< 50	181	42.5	27.08 (21.43–34.21)	<0.001	18.19 (13.80–23.97)	<0.001
50–99	101	13.6	8.70 (6.67–11.34)	<0.001	6.22 (4.66–8.31)	<0.001
100–199	119	3.9	2.47 (1.92–3.18)	<0.001	2.02 (1.55–2.62)	<0.001
200–349	122	1.6	1.00 (Reference)		1.00 (Reference)	
350–499	50	0.8	0.51 (0.37–0.71)	<0.001	0.48 (0.34–0.68)	<0.001
≥ 500	39	0.6	0.41 (0.28–0.58)	<0.001	0.46 (0.32–0.66)	<0.001
Total (Estimate per sqrt cell)	612	2.5	-0.242 ± 0.009		-0.213 ± 0.011	
					RR (≥ 500 v 350–499): 0.97 (0.62–1.50)	
**Progression of Disease**						
< 50	260	61.1	11.16 (9.43–13.22)	<0.001	6.84 (5.59–8.36)	<0.001
50–99	181	24.4	4.50 (3.74–5.41)	<0.001	3.19 (2.61–3.90)	<0.001
100–199	348	11.3	2.08 (1.79–2.42)	<0.001	1.84 (1.57–2.16)	<0.001
200–349	421	5.4	1.00 (Reference)		1.00 (Reference)	
350–499	193	3.1	0.56 (0.47–0.68)	<0.001	0.62 (0.51–0.75)	<0.001
≥ 500	113	1.8	0.34 (0.27–0.42)	<0.001	0.41 (0.33–0.52)	<0.001
Total (Estimate per sqrt cell)	1516	6.2	-0.176 ± 0.006		-0.139 ± 0.007	
					RR (≥ 500 v 350–499): 0.67 (0.51–0.87)	
**Grade 4 Events**						
< 50	212	49.8	7.38 (6.14–8.88)	<0.001	6.13 (5.01–7.49)	<0.001
50–99	164	22.1	3.24 (2.65–3.97)	<0.001	2.82 (2.27–3.50)	<0.001
100–199	351	11.4	1.71 (1.47–1.99)	<0.001	1.61 (1.37–1.88)	<0.001
200–349	510	6.6	1.00 (Reference)		1.00 (Reference)	
350–499	346	5.5	0.84 (0.73–0.97)	0.019	0.85 (0.73–0.98)	0.031
≥ 500	334	5.5	0.81 (0.69–0.94)	0.007	0.84 (0.71–0.98)	0.030
Total (Estimate per sqrt cell)	1917	7.9	-0.099 ± 0.007		-0.086 ± 0.007	
					RR (≥ 500 v 350–499): 0.99 (0.84–1.17)	
**Death, POD, and Grade 4**						
< 50	653	153.4	10.93 (9.64–12.40)	<0.001	7.88 (6.80–9.13)	<0.001
50–99	445	60.0	4.32 (3.77–4.95)	<0.001	3.39 (2.92–3.93)	<0.001
100–199	819	26.7	1.93 (1.73–2.15)	<0.001	1.75 (1.56–1.96)	<0.001
200–349	1054	13.5	1.00 (Reference)		1.00 (Reference)	
350–499	588	9.4	0.69 (0.62–0.78)	<0.001	0.72 (0.64–0.81)	<0.001
≥ 500	486	7.9	0.57 (0.50–0.65)	<0.001	0.64 (0.56–0.73)	<0.001
Total (Estimate per sqrt cell)	4045	16.6	-0.152 ± 0.005		-0.126 ± 0.006	
					RR (≥ 500 v 350–499): 0.89 (0.77–1.02)	

**Notes:** Relative rate estimates obtained using Poisson Regression

Adjusted for age, gender, history of AIDS or TB, and time updated viral load (<400, 400–9999, 10000+)

The difference in rates between the upper two CD4+ strata was not significant except for POD which was lower for those with latest CD4+ count of 500+ versus 350–499 cells/mm^3^ (crude rates were 1.8 versus 3.1 per 100 person years). The adjusted RRs and 95% CIs while the CD4+ count was 500+ versus 350–499 were 0.97 (95% CI: 0.62 to 1.50), 0.67 (95% CI: 0.51 to 0.87), 0.99 (95% CI: 0.84 to 1.17), and 0.89 (95% CI: 0.77 to 1.02), for death, POD, grade 4 events, and the composite outcome, respectively.

Table 4 summarizes event rates by latest CD4+ count and gives RRs by ART status using those with latest CD4+ cell count of 200–349 cells as reference. For the composite outcome, RRs for the upper two CD4+ strata compared to the 200–349 stratum were significantly less than 1.0 for those on and not on ART and rates in the upper two CD4+ strata did not differ from one another. The adjusted RRs (500+ versus 350–499) were 0.94 (95% CI: 0.81 to 1.10) and 0.79 (95% CI: 0.57 to 1.09) for those on and not on ART, respectively.

**Table 4:** Relative Rates of Morbidity and Mortality by Latest CD4 Count (cells/mm^3^) and ART Status: Morbidity and Mortality Cohort.

	On ART		Not on ART	
	Unadjusted	Adjusted ^1^	Unadjusted	Adjusted ^2^
***All-Cause Mortality***				
< 50	25.81 (19.94–33.42)	18.35 (13.24–25.43)	38.02 (21.12–68.44)	11.08 (5.31–23.12)
50–99	7.81 (5.80–10.52)	6.09 (4.38–8.47)	18.00 (10.22–31.67)	4.86 (2.34–10.09)
100–199	2.34 (1.76–3.09)	1.97 (1.47–2.64)	3.67 (2.02–6.66)	1.85 (0.91–3.74)
200–349	1.00 (reference)	1.00 (reference)	1.00 (reference)	1.00 (reference)
350–499	0.45 (0.30–0.66)	0.43 (0.28–0.64)	0.76 (0.40–1.43)	0.85 (0.42–1.67)
500 +	0.42 (0.28–0.63)	0.46 (0.31–0.70)	0.35 (0.15–0.81)	0.66 (0.30–1.46)
Slope (Square Root)	-0.242 ± 0.010	-0.218 ± 0.013	-0.254 ± 0.021	-0.147 ± 0.027
***Progression of Disease***				
< 50	11.95 (9.85–14.49)	7.32 (5.79–9.25)	12.71 (8.49–19.03)	6.82 (4.28–10.87)
50–99	4.54 (3.66–5.62)	3.28 (2.60–4.13)	7.11 (4.74–10.68)	3.27 (1.99–5.38)
100–199	2.30 (1.93–2.73)	1.98 (1.65–2.37)	2.04 (1.46–2.87)	1.55 (1.08–2.23)
200–349	1.00 (reference)	1.00 (reference)	1.00 (reference)	1.00 (reference)
350–499	0.61 (0.49–0.77)	0.68 (0.54–0.86)	0.49 (0.35–0.69)	0.56 (0.39–0.79)
500 +	0.38 (0.29–0.49)	0.46 (0.35–0.59)	0.26 (0.16–0.41)	0.44 (0.28–0.69)
Slope (Square Root)	-0.177 ± 0.007	-0.139 ± 0.008	-0.194 ± 0.013	-0.133 ± 0.016
***Grade 4 Events***				
< 50	6.98 (5.74–8.50)	5.01 (4.00–6.26)	9.21 (5.49–15.45)	8.50 (4.89–14.76)
50–99	3.08 (2.48–3.82)	2.44 (1.93–3.09)	4.21 (2.32–7.62)	3.08 (1.47–6.45)
100–199	1.65 (1.41–1.94)	1.47 (1.24–1.74)	1.86 (1.20–2.89)	1.74 (1.09–2.78)
200–349	1.00 (reference)	1.00 (reference)	1.00 (reference)	1.00 (reference)
350–499	0.84 (0.71–0.98)	0.86 (0.73–1.01)	0.86 (0.61–1.20)	0.93 (0.64–1.33)
500 +	0.87 (0.73–1.02)	0.90 (0.76–1.07)	0.55 (0.37–0.83)	0.73 (0.47–1.13)
Slope (Square Root)	-0.093 ± 0.007	-0.070 ± 0.008	-0.127 ± 0.015	-0.107 ± 0.017
***Death*, *POD*, *and Grade 4 Events***				
< 50	10.73 (9.37–12.30)	7.43 (6.29–8.77)	13.79 (9.67–19.66)	8.08 (5.49–11.89)
50–99	4.10 (3.53–4.76)	3.19 (2.71–3.76)	6.99 (4.95–9.85)	3.48 (2.28–5.29)
100–199	1.94 (1.73–2.19)	1.71 (1.51–1.94)	2.12 (1.62–2.78)	1.67 (1.25–2.22)
200–349	1.00 (reference)	1.00 (reference)	1.00 (reference)	1.00 (reference)
350–499	0.71 (0.63–0.81)	0.75 (0.66–0.85)	0.66 (0.51–0.84)	0.72 (0.56–0.93)
500 +	0.64 (0.56–0.74)	0.70 (0.61–0.81)	0.38 (0.28–0.50)	0.57 (0.42–0.77)
Slope (Square Root)	-0.147 ± 0.006	-0.118 ± 0.007	-0.181 ± 0.011	-0.129 ± 0.012

**Notes:** ^1^ Adjusted for age, gender, history of AIDS or TB, and time-updated viral load (<400, 400–999, 1000+).

^2^Adjusted for age, gender, history of AIDS or TB, and time-updated viral load (log10).

S3 Table compares results from a cause-specific hazard and competing risk proportional hazards regression model for mortality and POD for those not on ART. CD4+ slopes and HR estimates for counts less than 200 cells are smaller for the competing risk regression models than the cause-specific hazards model. Estimates for counts at 350–499 and 500+ cells versus 200–349 cells are similar for the two models, likely as a result of less dependence between the competing events of death and POD with initiation of ART at higher counts.

We also assessed whether rates of morbidity and mortality differed between those on and not on ART according to latest CD4+ cell count level (Table 5). Overall, for the composite outcome, the adjusted RR (ART/no ART) was 0.83 (95% CI: 0.74–0.93). This lower overall rate for those on ART compared to those not on ART primarily resulted from a significantly lower rates on ART in the lower two CD4+ strata. For the upper two latest CD4+ strata, the RR (ART/no ART) was 1.05 (95% CI: 0.86 to 1.28).

**Table 5:** Morbidity and Mortality Rates by Latest CD4 Count and ART Status and Relative Rates (ART vs No ART) by CD4 Count (cells/mm^3^): Morbidity and Mortality Cohort.

	On ART		Not on ART		Relative Rate (ART vs No ART)	
	No. Events	Event Rate Per 100 PY	No. Events	Event Rate Per 100 PY	Unadjusted	Adjusted ^1^
***All-Cause Mortality***						
< 200	339	8.9	62	14.7	0.60 (0.46–0.80)	0.92 (0.64–1.32)
200–349	95	1.6	27	1.6	1.01 (0.66–1.54)	0.85 (0.55–1.33)
350–499	35	0.7	15	1.2	0.59 (0.32–1.08)	0.56 (0.28–1.15)
500 +	32	0.7	7	0.5	1.20 (0.53–2.72)	1.03 (0.44–2.43)
Total ^2^	501	2.5	111	2.4	0.72 (0.58–0.90)	0.85 (0.66–1.09)
***Progression of Disease***						
< 200	652	17.1	137	32.5	0.52 (0.43–0.64)	0.52 (0.42–0.66)
200–349	280	4.6	141	8.1	0.57 (0.45–0.71)	0.47 (0.37–0.61)
350–499	142	2.8	51	4.0	0.71 (0.50–1.02)	0.74 (0.50–1.11)
500 +	86	1.8	27	2.1	0.84 (0.52–1.36)	0.73 (0.44–1.22)
Total ^2^	1,160	5.9	356	7.6	0.58 (0.51–0.67)	0.55 (0.47–0.64)
***Grade 4 Events***						
< 200	652	17.1	75	17.8	0.98 (0.75–1.28)	0.94 (0.70–1.26)
200–349	408	6.7	102	5.9	1.17 (0.90–1.50)	1.12 (0.86–1.47)
350–499	283	5.7	63	5.0	1.14 (0.85–1.54)	1.10 (0.80–1.51)
500 +	292	6.0	42	3.3	1.83 (1.26–2.64)	1.64 (1.12–2.40)
Total ^2^	1,635	8.3	282	6.0	1.20 (1.03–1.39)	1.15 (0.98–1.34)
***Death*, *POD*, *and Grade 4 Events***						
< 200	1,643	43.1	274	65.0	0.68 (0.57–0.81)	0.73 (0.60–0.88)
200–349	784	13.0	270	15.5	0.84 (0.71–1.00)	0.75 (0.63–0.90)
350–499	459	9.2	129	10.1	0.91 (0.72–1.16)	0.91 (0.70–1.18)
500 +	410	8.5	76	5.9	1.42 (1.07–1.88)	1.28 (0.94–1.73)
Total ^2^	3,296	16.7	749	15.9	0.84 (0.76–0.94)	0.83 (0.74–0.93)

**Notes:** ^1^ Adjusted for age, gender, and history of AIDS or TB.

^2^ Unadjusted relative rates pooled within CD4 category.

Differences between those on and not on ART varied for the different components of the composite outcome. For mortality, the adjusted RR (ART/no ART) was 0.85 (95% CI; 0.66–1.09). POD rates were lower for those on ART as compared to those not on ART in each CD4+ stratum; overall the adjusted RR was 0.55 (95% CI: 0.47–0.64). The most common POD event was pulmonary TB. Of the 1,160 POD events that occurred on ART, 747 (64.4%) were due to pulmonary TB; 246 of the 356 POD events (69.1%) that occurred among participants not taking ART were attributed to pulmonary TB.

For grade 4 events, overall, the adjusted RR (ART/no ART) was 1.15 (95% CI: 0.98–1.34). Grade 4 events accounted for approximately 50% of the morbidity among participants taking ART (1,635 of 3,296 events). For those not on ART, most events were due to POD and grade 4 events comprised 38% of all events.

Tables 6 and 7 examine the types of grade 4 events by latest CD4+ count and ART status. Overall, few serious non-AIDS events by our definition (see Methods) occurred. Among participants on ART, there were 89 serious non-AIDS events (rate = 0.5 per 100 person years); 14 events occurred among participants who were not taking ART (rate = 0.3 per 100 person years). The adjusted RR (ART/no ART) was 1.48 (95% CI: 0.78 to 2.79).

**Table 6:** Grade 4 Event Types by Latest CD4 Count (cells/mm^3^) and ART Status: Morbidity and Mortality Cohort.

	On ART		Not On ART		Relative Rate (ART vs No ART)	
***Serious Non-Aids Events***	No. Events	Event Rate Per 100 PY	No. Events	Event Rate Per 100 PY	Unadjusted	Adjusted^1^
<200	35	0.9	3	0.7	1.36 (0.41–4.47)	3.65 (0.47–28.26)
200–349	22	0.4	4	0.2	1.68 (0.55–5.14)	1.96 (0.54–7.07)
350–499	16	0.3	2	0.2	1.99 (0.45–8.84)	1.23 (0.27–5.63)
≥ 500	16	0.3	5	0.4	0.84 (0.30–2.36)	0.64 (0.23–1.80)
Total	89	0.5	14	0.3	1.37 (0.77–2.45)	1.48 (0.78–2.79)
***Infections***						
<200	130	3.4	18	4.3	0.81 (0.49–1.33)	0.82 (0.47–1.43)
200–349	69	1.1	19	1.1	1.03 (0.62–1.73)	1.00 (0.56–1.76)
350–499	42	0.8	12	0.9	0.86 (0.45–1.67)	0.78 (0.38–1.61)
≥ 500	38	0.8	5	0.4	1.99 (0.68–5.86)	2.33 (0.65–8.28)
Total	279	1.4	54	1.1	1.00 (0.74–1.36)	1.00 (0.71–1.40)
***Gastro***						
<200	123	3.2	13	3.1	1.00 (0.56–1.82)	0.95 (0.49–1.86)
200–349	60	1.0	12	0.7	1.42 (0.70–2.90)	1.62 (0.70–3.73)
350–499	39	0.8	5	0.4	2.07 (0.81–5.28)	1.77 (0.65–4.80)
≥ 500	25	0.5	4	0.3	1.54 (0.53–4.48)	1.20 (0.42–3.43)
Total	247	1.3	34	0.7	1.37 (0.91–2.05)	1.34 (0.85–2.10)
***Injuries***						
<200	46	1.2	1	0.2	5.06 (0.70–36.42)	4.19 (0.58–30.19)
200–349	47	0.8	14	0.8	0.96 (0.53–1.74)	0.95 (0.48–1.85)
350–499	44	0.9	10	0.8	1.12 (0.56–2.20)	1.14 (0.56–2.33)
≥ 500	36	0.7	2	0.2	4.73 (1.15–19.52)	5.02 (1.23–20.53)
Total	173	0.9	27	0.6	1.46 (0.97–2.18)	1.49 (0.96–2.30)

**Notes:** Relative rate estimates obtained using Poisson Regression

^1^Adjusted for age, gender, and history of POD. Unadjusted relative rates for total pooled within CD4 category

**Table 7:** Rates of Grade 4 Event Types by Latest CD4 Count (cells/mm^3^) and ART Status: Morbidity and Mortality Cohort.

	On ART		Not On ART		Relative Rate (ART vs No ART)	
***Nervous System***	No. Events	Event Rate Per 100 PY	No. Events	Event Rate Per 100 PY	Unadjusted	Adjusted^1^
<200	58	1.5	8	1.9	0.81 (0.38–1.75)	0.69 (0.32–1.48)
200–349	43	0.7	10	0.6	1.31 (0.52–3.26)	1.09 (0.43–2.78)
350–499	34	0.7	11	0.9	0.80 (0.36–1.75)	0.80 (0.32–1.97)
≥ 500	33	0.7	4	0.3	2.10 (0.57–7.66)	1.79 (0.48–6.60)
Total	168	0.9	33	0.7	1.12 (0.70–1.77)	1.02 (0.63–1.65)
***Psychiatric***						
<200	51	1.3	3	0.7	1.86 (0.57–6.05)	1.48 (0.43–5.09)
200–349	34	0.6	11	0.6	0.90 (0.43–1.87)	0.84 (0.40–1.75)
350–499	31	0.6	5	0.4	1.62 (0.60–4.43)	1.43 (0.51–4.00)
≥ 500	19	0.4	7	0.5	0.70 (0.30–1.65)	0.60 (0.24–1.55)
Total	135	0.7	26	0.6	1.14 (0.73–1.77)	0.98 (0.62–1.55)
***Metabolic***						
<200	59	1.5	3	0.7	2.17 (0.68–6.96)	2.74 (0.65–11.48)
200–349	46	0.8	2	0.1	6.62 (1.60–27.32)	6.37 (1.54–26.29)
350–499	24	0.5	3	0.2	2.15 (0.64–7.28)	1.98 (0.57–6.82)
≥ 500	24	0.5	4	0.3	1.53 (0.53–4.45)	1.50 (0.52–4.35)
Total	153	0.8	12	0.3	2.73 (1.48–5.02)	2.61 (1.40–4.90)
***All Others***						
<200	150	3.9	25	5.9	0.67 (0.42–1.07)	0.62 (0.37–1.04)
200–349	92	1.5	30	1.7	0.90 (0.54–1.50)	0.86 (0.51–1.47)
350–499	55	1.1	16	1.3	0.87 (0.49–1.56)	0.88 (0.46–1.70)
≥ 500	106	2.2	12	0.9	2.38 (1.20–4.70)	1.85 (0.93–3.67)
Total	403	2.0	83	1.8	1.02 (0.77–1.34)	0.94 (0.70–1.27)

**Notes:** Relative rate estimates obtained using Poisson Regression

^1^Adjusted for age, gender, and history of POD. Unadjusted relative rates for total pooled within CD4 category

For 6 SOCs, over 100 events were reported. With the exception of events which were coded as metabolic events, the overall (pooled over CD4+ strata) differences between those on and not on ART were not significant. Among patients taking ART, 153 metabolic events occurred (rate = 0.8 per 100 person years) versus 12 events (rate = 0.3 per 100 person years) among participants not on ART (adjusted RR = 2.61; 95% 1.40–4.90). Among patients on ART the most common metabolic grade 4 events were diabetes (29 events), hyperlactacidemia (28),dehydration (18), hyperglycemia (15), and lactic acidosis (13). The excess events among participants on ART compared to those not on ART was greatest at CD4+ counts < 350 cells.

## Discussion

We studied a large cohort of HIV positive participants in South Africa who were in the SANDF or who were a dependent of a SANDF member. Our main findings are: 1) death, POD and grade 4 event rates were markedly higher in the CD4+ count strata <200; 2) overall, and for those on and not on ART, differences in mortality and serious morbidity between those with CD4+ count 350–499 and 500+ were small and not significant; and 3) the type of morbidity experienced varied according to use of ART and latest CD4+ cell count.

Our finding is consistent with large cohorts from Western countries and from Africa and Asia [3–5,10–12]. Death rates and AIDS rates increased sharply the lower the latest CD4+ cell count. Risk gradients are evident among both ART-naïve participants and among those receiving ART. In a competing risk regression analysis we found that the strength of the associations between latest CD4+ cell count and mortality and POD for those not on ART is overestimated particularly at lower latest CD4+ cell count levels. At higher counts (350+ cells), differences relative to those with counts 200–349 cells are similar for the cause-specific hazard analysis and the competing risk analysis.

Data on the relationship of non-AIDS morbidity and latest CD4+ cell counts is more limited. In the EuroSIDA cohort in which most participants were taking ART and who had a nadir CD4+ cell count of 178 cells, non-AIDS events that included malignancies, major CVD events, pancreatitis, major liver disease events and end-stage renal disease were associated with latest CD4+ cell count but the association was weaker than the association of latest CD4+ count and AIDS [14]. Also, in EuroSIDA, there was not a significant difference in non-AIDS rates for those with counts of 500 cells or higher versus those with counts 351–500 cells. In Phidisa, there were relatively few serious non-AIDS events, but with consideration of any grade 4 event, like EuroSIDA, CD4+ risk gradients were not as large as for AIDS. Also, at higher CD4+ counts, grade 4 event rates did not differ significantly between those with latest CD4+ counts of 500 cells or higher versus those with counts 350–399 cells for either those on or not on ART. In Phidisa, the median CD4+ cell count at ART initiation was 143 cells, 25 cells lower than the EuroSIDA nadir CD4+ count.

The question about safe deployment primarily relates to those with CD4+ count above 350 cells so we focused many of our comparisons and our review of the literature on that group. Two large cohort studies have assessed the risk of death and AIDS at latest CD4+ cell counts above 350 cells. In a cohort study of ART-naïve participants enrolled at North American and European sites, death rates for participants with latest counts of 500–699 and 700+ cells were 23% and 34% lower than those with latest counts of 350–499 cells [29]. In a collaboration of European cohorts, that studied participants on ART with a suppressed viral load, rates of AIDS or death and of death were lower for those with latest counts greater than or equal to 500 cells as compared to 350–499. AIDS or death event rates were 0.52 and 0.79 per 1,000 person years for these two CD4+ strata; death rates were 0.24 and 0.38 per 1,000 person years, respectively [30]. In Phidisa, death rates were similar for those with latest CD4+ counts of 500 or higher as compared to those with counts 350–499 cells; however, the numbers of deaths in these strata were not large, particularly when further categorized by use of ART, and as a consequence, confidence intervals for the relative risks were wide both for the mortality cohort (95% CI: 0.70–1.46) and for the morbidity and mortality cohort (95% CI: 0.62–1.50). In the Phidisa morbidity and mortality cohort, POD (AIDS or pulmonary TB) rates were significantly lower for those with counts of 500 cells or higher compared to those with latest CD4+ counts 350–499; however, the absolute rate differences were small (3.1 versus 1.8, overall; 2.8 versus 1.8 for those on ART; and 4.0 versus 2.1 per 100 person years for those not on ART).

In resource-limited countries, there are fewer cohort studies that compare rates of mortality and AIDS among participants in latest CD4+ strata above 350 cells. Lewden summarized death rates by latest CD4+ cell count for ART-naïve participants enrolled in five cohorts in West Africa, and concluded that rates varied across cohorts, likely as a result of lost to follow-up differences, and were higher than rates reported for high income countries [10]. In their study, rates of death were 3.0, 1.5, and 0.3 per 100 person years for those with latest CD4+ counts 201–350, 351–500, and 501+ cells/mm^3^, respectively. Overall, there were only 80 deaths in this study and for the upper two CD4+ strata there were only 23 and 7 deaths, respectively. Nevertheless, comparisons with two large European cohorts do suggest that CD4+ specific rates are higher in the West African study. In the CASCADE study, the rates (number of deaths) were 0.92 (47), 0.61 (50), and 0.37 (57) [11] for those with CD4+ count 200–349, 350–499, and 500+ cells, respectively; in the UK CHIC study, these rates (number of deaths) were 0.58 (46), 0.32 (30) and 0.18 (22) [5]. In the Phidisa mortality cohort, corresponding mortality rates (number of deaths) for ART-naïve participants were also greater than in CASCADE and UK CHIC and more similar to the cohorts in West Africa. Death rates were 3.2 (131), 1.4 (40), and 1.1 (30) for those with latest CD4+ counts of 200–349, 350–499 and 500+ cells, respectively. For the morbidity and mortality cohort of Phidisa, these CD4+ specific mortality rates per 100 person years (number of deaths) were 1.6 (27), 1.2 (15), and 0.5 (7). The lower mortality rate for the morbidity and mortality cohort compared to the mortality cohort could be due to chance or to the greater availability and uptake of ART with time as has been described in other settings in Africa [31].

Among participants on ART, CD4+ specific mortality rates are also lower in the CASCADE study as compared to cohorts in resource-limited settings and to Phidisa. In CASCADE, death rates (number of deaths) for participants on ART according to latest CD4+ counts of 200–349, 350–499, and 500+ cells were 0.84 (22), 0.42 (15), and 0.30 (25), respectively. Among participants receiving ART in cohorts in sub-Saharan Africa and Asia, rates were 1.8, 0.9, and 0.3 per 100 person years for these 3 strata [12]. For a cohort study in Cape Town, South Africa, mortality rates were 2.0, 2.0, and 1.2 for those with latest CD4+ counts 300–399, 400–499, and 500+ cells, respectively [9]. Like Phidisa, in both of these cohorts ART was initiated for most participants at CD4+ counts < 200 cells and the majority of deaths on ART occurred among participants with latest CD4+ counts < 200 cells. In the mortality cohort of Phidisa, death rates (number of deaths) for those on ART with latest CD4+ counts of 200–349, 350–499, and 500+ cells were 1.6 (99), 0.7 (35), and 0.7 (33), respectively. The CD4+ specific death rates above 200 cells were similar for the morbidity and mortality cohort of Phidisa and for participants enrolled in Phidisa 2 and for those who were not. However, in the lowest latest CD4+ count group (<50) considered, the death rate in Phidisa 2 was more than double that for participants who were on ART but not in Phidisa 2. We attribute this to the fact that more participants with advanced HIV were enrolled in Phidisa 2; as previously reported, over one-half of the deaths during the trial occurred in the first 6 months of follow-up [25].

In Phidisa, latest CD4+ specific death rates were greater for those not on ART as compared to those on ART. In the CASCADE cohort, death rates on and not on ART according to latest CD4+ specific strata have been compared, and like the death rates for the Phidisa mortality cohort, rates were lower for those on ART compared to those who were not on ART [11]. For participants in the Phidisa morbidity and mortality cohort, POD rates were lower for those on ART within each latest CD4+ count stratum; grade 4 rates tended to be higher. When only the upper two CD4+ strata were considered, the difference between the ART and no ART groups for the composite outcome were small and not significantly different from one another.

As described in other cohorts, morbidity at higher CD4+ counts is dominated by non-AIDS events [14, 16–18, 32]. Many of these events are more life-threatening than AIDS [19, 27]. Non-AIDS events occur among participants with suppressed viral load and risk increases with advancing age [33]. The greater rate of grade 4 events as compared to AIDS event in Phidisa is most evident for those on ART. While the relative contributions of HIV and ART treatment for HIV to the risk of non-AIDS morbidity is uncertain and is currently being examined in a large clinical trial, it appears that chronic inflammation due to HIV, that remains present even with suppressive ART, as well as some ART, may be related to an increased risk of death and some serious non-AIDS conditions [34–38].

There are several strengths of our study. Foremost is the long-term follow-up of HIV participants for mortality, AIDS events and non-AIDS events. A second strength is the low lost to follow-up rate, particularly in the morbidity and mortality cohort. Several other studies have reported higher rates of lost to follow-up both for patients beginning ART and in pre-ART programs [12, 39–43]. This has been a major problem of many HIV treatment programs. We attribute the excellent follow-up in Phidisa to close, regular monitoring of performance at each site, the use of appointment reminders and the integration of HIV care at the SAMHS sites. A third strength is the ability to assess outcomes related to latest CD4+ cell counts during follow-up while on and not on ART in the same cohorts of participants as in the CASCADE study [11]. To our knowledge this is the only African cohort in which this has been possible to date.

A weakness is that for many of the POD events confirmatory diagnoses were not made. A second weakness is the composite outcome of death, POD or grade 4 events, includes events of varying severity. This is a general problem with composite outcomes. Future research will be aimed at understanding the risk of death of different POD and grade 4 events. Finally, the data available do not allow us to determine whether participants were soldiers in SANDF or their family members.

In summary, these findings which take into account serious morbidity as well as mortality on and not on ART should allow policymakers to make a more informed decision on the deployment of HIV positive soldiers. Further research is needed to determine what fraction of the morbidity and mortality observed at higher CD4+ cell counts in this cohort while on ART is a consequence of beginning ART very late, the type of ART used, or underlying conditions in the population.

## Supporting Information

S1 Table Enrollment characteristics of Phidisa HIV positive participants by protocol enrollment: mortality cohort. (DOCX)

S2 Table Characteristics of Phidisa HIV positive participants by mortality status: morbidity and mortality cohort. (DOCX)

S3 Table Mortality and Progression of Disease by Latest CD4+ Cell Count by Use of ART: A Comparison between a Cause-Specific Hazard and Competing Risk Proportional Hazards Regression Model. (DOCX)
