# Supplementary material for: Morbidity and Mortality According to Latest CD4+ Cell Count among HIV Positive Individuals in South Africa Who Enrolled in Project Phidisa
Source: PLoS One. 2015 Apr 9;10(4):e0121843. doi: 10.1371/journal.pone.0121843 (PMC4391777; doi:10.1371/journal.pone.0121843)
Supplement: S1 Table — (DOCX) [file pone.0121843.s001.docx]

| **S1 S1. Characteristics at Enrolment of Phidisa HIV Positive Participants by Protocol Enrollment: Mortality Cohort** | | | | |
| --- | --- | --- | --- | --- |
| ***Demographics*** | **Phidisa 1** | **Phidisa 2** | **Phidisa 1A** | **Total** |
| Age (median years) | 34.0 [31.0, 38.0] | 34.0 [31.0, 38.0] | 38.0 [34.0, 41.0] | 35.0 [32.0, 39.0] |
| Female (%) | 1372 (40.1%) | 567 (32.0%) | 727 (37.8%) | 2666 (37.5%) |
| Location of home (% rural) | 1349 (39.5%) | 755 (42.6%) | 875 (45.5%) | 2979 (41.9%) |
| Marital status (% married) | 2210 (64.8%) | 1104 (62.3%) | 1313 (68.2%) | 4627 (65.1%) |
| Education (% HS or tertiary) | 2801 (82.2%) | 1521 (86.0%) | 1441 (75.1%) | 5763 (81.2%) |
| Body Mass Index (median kg/m^2^) | 23.8 [20.9, 27.8] | 22.9 [20.4, 25.9] | 24.7 [21.5, 28.7] | 23.8 [20.9, 27.6] |
| On ART at Baseline (%) | 134 (4.8%) | 1 (0.1%) | 147 (8.1%) | 282 (4.4%) |
| ***HIV characteristics*** |  |  |  |  |
| CD4 count (median cell/mm^3^) | 288.0 [145.0, 449.0] | 113.0 [48.0, 176.0] | 215.5 [93.0, 349.0] | 207.0 [87.0, 360.0] |
| < 50 | 421 (12.4%) | 456 (26.0%) | 277 (14.4%) | 1154 (16.3%) |
| 50-99 | 252 (7.4%) | 308 (17.5%) | 228 (11.9%) | 788 (11.1%) |
| 100-199 | 398 (11.7%) | 692 (39.4%) | 399 (20.8%) | 1489 (21.0%) |
| 200-349 | 998 (29.4%) | 244 (13.9%) | 539 (28.0%) | 1781 (25.2%) |
| 350-499 | 653 (19.2%) | 52 (3.0%) | 252 (13.1%) | 957 (13.5%) |
| 500 + | 675 (19.9%) | 5 (0.3%) | 227 (11.8%) | 907 (12.8%) |
| HIV viral load (median log_10_ copies/mL) | 4.5 [3.8, 5.2] | 5.1 [4.7, 5.4] | 4.8 [3.9, 5.3] | 4.8 [4.1, 5.3] |
| Hb (median g/dl) | 13.0 [11.3, 14.5] | 12.7 [11.3, 14.0] | 12.9 [11.2, 14.3] | 12.9 [11.3, 14.3] |
| ***Co-morbidities*** |  |  |  |  |
| Hepatitis B - SAG positive (%) | 121 (3.6%) | 78 (4.4%) | 58 (3.0%) | 257 (3.6%) |
| Hepatitis C (%) | 18 (0.5%) | 13 (0.7%) | 6 (0.3%) | 37 (0.5%) |
| History of AIDS or Pulmonary/Extrapulmonary TB (%) | 603 (21.4%) | 566 (32.0%) | 440 (24.3%) | 1609 (25.2%) |
| ***Number of patients*** | **3418** | **1771** | **1925** | **7114** |
| **Notes:** | | | | |
