# Supplementary material for: Morbidity and Mortality According to Latest CD4+ Cell Count among HIV Positive Individuals in South Africa Who Enrolled in Project Phidisa
Source: PLoS One. 2015 Apr 9;10(4):e0121843. doi: 10.1371/journal.pone.0121843 (PMC4391777; doi:10.1371/journal.pone.0121843)
Supplement: S2 Table — (DOCX) [file pone.0121843.s002.docx]

| **S2 Table. Characteristics at Enrolment of Phidisa HIV Positive Participants by Mortality Status: Morbidity and Mortality Cohort** | | | | |
| --- | --- | --- | --- | --- |
| ***Demographics*** | **Known Deceased** | **Known Alive** | **Unknown** | **Total** |
| Age (median years) | 36.0 [33.0, 40.0] | 35.0 [32.0, 39.0] | 34.0 [30.0, 38.0] | 35.0 [32.0, 39.0] |
| Female (%) | 147 (23.6%) | 1944 (39.3%) | 183 (45.0%) | 2274 (38.1%) |
| Location of home (% rural) | 261 (41.8%) | 2183 (44.2%) | 132 (32.4%) | 2576 (43.1%) |
| Marital status (% married) | 374 (59.9%) | 3336 (67.5%) | 266 (65.4%) | 3976 (66.6%) |
| Education (% HS or tertiary) | 470 (75.3%) | 3998 (81.0%) | 343 (84.7%) | 4811 (80.7%) |
| Body Mass Index (median kg/m^2^) | 21.7 [19.2, 24.9] | 24.3 [21.5, 28.1] | 24.3 [21.1, 28.1] | 24.1 [21.1, 27.9] |
| On ART at Baseline (%) | 12 (2.1%) | 219 (4.7%) | 13 (3.5%) | 244 (4.4%) |
| ***HIV characteristics*** |  |  |  |  |
| CD4 count (median cell/mm^3^) | 98.0 [29.5, 225.0] | 218.0 [105.0, 370.0] | 225.0 [119.0, 379.0] | 207.0 [94.0, 359.0] |
| < 50 | 208 (33.5%) | 644 (13.1%) | 39 (9.6%) | 891 (15.0%) |
| 50-99 | 107 (17.3%) | 517 (10.5%) | 38 (9.4%) | 662 (11.1%) |
| 100-199 | 130 (21.0%) | 1108 (22.5%) | 102 (25.2%) | 1340 (22.5%) |
| 200-349 | 102 (16.5%) | 1291 (26.2%) | 106 (26.2%) | 1499 (25.2%) |
| 350-499 | 41 (6.6%) | 687 (14.0%) | 61 (15.1%) | 789 (13.3%) |
| 500 + | 32 (5.2%) | 675 (13.7%) | 59 (14.6%) | 766 (12.9%) |
| HIV viral load (median log_10_ copies/mL) | 5.2 [4.7, 5.6] | 4.7 [4.0, 5.2] | 4.6 [3.9, 5.2] | 4.8 [4.1, 5.3] |
| Hb (median g/dl) | 12.1 [10.5, 13.7] | 13.1 [11.6, 14.4] | 12.9 [11.4, 14.4] | 13.0 [11.4, 14.4] |
| ***Co-morbidities*** |  |  |  |  |
| Hepatitis B - SAG positive (%) | 29 (4.7%) | 173 (3.5%) | 4 (1.0%) | 206 (3.5%) |
| Hepatitis C (%) | 2 (0.3%) | 26 (0.5%) | 3 (0.7%) | 31 (0.5%) |
| History of AIDS or Pulmonary/Extrapulmonary TB (%) | 238 (41.5%) | 1067 (22.8%) | 75 (20.4%) | 1380 (24.5%) |
| ***Number of patients*** | **624** | **4945** | **407** | **5976** |
| **Notes:** | | | | |
