# Supplementary material for: Morbidity and Mortality According to Latest CD4+ Cell Count among HIV Positive Individuals in South Africa Who Enrolled in Project Phidisa
Source: PLoS One. 2015 Apr 9;10(4):e0121843. doi: 10.1371/journal.pone.0121843 (PMC4391777; doi:10.1371/journal.pone.0121843)
Supplement: S3 Table — (DOCX) [file pone.0121843.s003.docx]

| **S3 Table. Mortality and Progression of Disease by Latest CD4+ Cell Count: A Comparison Between Cause-Specific Hazard and Competing Risk Proportional Hazards Regression Model** | | |
| --- | --- | --- |
| ***Mortality*** | Cause-Specific Hazard HR (95% CI) | Competing Risk Hazard HR (95% CI) |
| < 50 cells | 20.94 (11.61-37.78) | 5.11 (3.00-8.71) |
| 50-99 | 13.19 (7.13-24.04) | 4.03 (2.21-7.37) |
| 100-199 | 2.87 (1.58-5.22) | 1.36 (0.74-2.48) |
| 200-349 | 1.00 (reference) | 1.00 (reference) |
| 350-499 | 0.80 (0.43-1.51) | 0.84 (0.44-1.57) |
| ≥ 500 | 0.36 (0.16-0.84) | 0.45 (0.19-1.03) |
| Slope (Square Root) | -0.21 ± 0.02 | -0.12 ± 0.02 |
| ***Progression of Disease*** |  |  |
| < 50 cells | 8.03 (5.59-11.53) | 1.92 (1.38-2.67) |
| 50-99 | 5.92 (4.17-8.55) | 1.83 (1.31-2.56) |
| 100-199 | 1.97 (1.48-2.63) | 0.98 (0.73-1.32) |
| 200-349 | 1.00 (reference) | 1.00 (reference) |
| 350-499 | 0.58 (0.43-0.78) | 0.58 (0.43-0.79) |
| ≥ 500 | 0.26 (0.18-0.40) | 0.31 (0.20-0.43) |
| Slope (Square Root) | -0.16 ± 0.01 | -0.08 ± 0.01 |
| **Notes:**  Both models are unadjusted models | | |
|  | | |
